# Long‐term impact of postoperative pneumonia after curative gastrectomy for elderly gastric cancer patients

**DOI:** 10.1002/ags3.12037

**Published:** 2017-09-19

**Authors:** Satoshi Suzuki, Shingo Kanaji, Yoshiko Matsuda, Masashi Yamamoto, Hiroshi Hasegawa, Kimihiro Yamashita, Taro Oshikiri, Takeru Matsuda, Yasuo Sumi, Tetsu Nakamura, Yoshihiro Kakeji

**Affiliations:** ^1^ Division of Gastrointestinal Surgery Department of Surgery Graduate School of Medicine Kobe University Kobe Hyogo Japan

**Keywords:** elderly, gastrectomy, gastric cancer, pneumonia, prognosis

## Abstract

With the extension of life expectancy, cancer has been increasing in elderly populations. Postoperative pneumonia can negatively influence immediate mortality following gastrectomy for elderly patients, but its impact on long‐term survival remains unclear. We retrospectively reviewed the cases of patients aged ≥75 years who underwent curative gastrectomy for gastric cancer from 2000 to 2014 to determine the long‐term effects of postoperative pneumonia and to identify independent risk factors along with physical status and surgical procedure. Of 250 patients, 32 (12.8%) developed postoperative pneumonia. Patients with postoperative pneumonia had significantly worse overall survival (OS) than those without postoperative pneumonia (*P<*.001). A multivariate analysis identified postoperative pneumonia as a prognostic factor for OS (hazard ratio, 2.06; 95% CI, 1.05‐3.75; *P*=.036). Significant risk factors for the development of postoperative pneumonia were male gender (*P*=.026) and D2 lymphadenectomy (*P*<.001). D2 lymphadenectomy was associated with poorer OS than D1 or D1+lymphadenectomy in patients with an American Society of Anesthesiologists (ASA) score 3 (*P*=.026), but did not influence OS negatively in patients with an ASA score ≤2. Limited lymphadenectomy did not affect the cancer‐specific survival of elderly patients with ASA score 3. Postoperative pneumonia following gastrectomy has an adverse impact on the long‐term survival of elderly gastric cancer patients. A limited lymphadenectomy during curative resection should be considered to prevent postoperative pneumonia in frail elderly patients with ASA score 3. Postoperative pneumonia following gastrectomy has an adverse impact on the long‐term survival of elderly gastric cancer patients. Extent of lymph node dissection during curative resection should be limited to prevent postoperative pneumonia, based on the patient's frailty.

## 1 INTRODUCTION

The populations of many countries are aging rapidly and, in Japan, it is estimated that 26% of the population is now ≥65 years old.^1^ The number of elderly patients with gastric cancer has also increased annually and, in 2011, 27% of gastric cancer patients in Japan were over 80 years old.^2^ Development of therapeutic strategies for this segment of the population presents a challenge, as elderly patients usually have comorbidities and poor physical status that adversely affect their surgical outcomes and prolong their postoperative disabilities.^3^, ^4^, ^5^

Surgical resection is the main therapeutic modality for malignancies, but surgery has the potential to result in significant worsening of an elderly patient's quality of life. Postoperative complications also seriously impair physical status and directly result in poor outcomes in elderly patients.^6^ These disadvantages of surgery can worsen the prognosis of elderly patients per se. Surgical treatment must be planned on the basis of tolerability, curability and future quality of life. We need to understand the causes of reduced survival in order to improve the surgical outcomes of elderly patients.

Pneumonia is one of the main causes of death in the elderly,^7^ and it is a relatively common postoperative complication. Among abdominal surgeries, gastrectomy is especially associated with postoperative pneumonia, with the second highest incidence (16‐17%) following thoracic surgery (15‐37.5%).^8^, ^9^ Several studies have reported that pulmonary complications negatively affect not only short‐term mortality but also long‐term survival after thoracic surgeries such as resection of esophageal and lung cancer.^10^, ^11^, ^12^, ^13^ Although a previous study reported that patients with postoperative pneumonia following gastrectomy had high postoperative mortality,^14^ the potential long‐term impact of postoperative pneumonia has not been investigated. Identification of risk factors for postoperative pneumonia may have a crucial role in the development of therapeutic strategies to reduce the incidence and impact of pneumonia in elderly patients.

We conducted the present study to evaluate the impact of postoperative pneumonia after curative gastrectomy on long‐term survival in elderly gastric cancer patients and to identify potential independent risk factors.

## 2 MATERIALS AND METHODS

### 2.1 Patients and data retrieval

From January 2000 to December 2014, a total of 1071 patients underwent gastrectomy for gastric cancer at Kobe University Hospital. Among them, the cases of 250 patients aged ≥75 years who underwent curative resection (R0 resection) were analyzed in this retrospective study. Patients who underwent surgery for remnant gastric cancer or who received neoadjuvant chemotherapy were excluded.

Clinical characteristics, surgical outcomes, pathological findings and follow‐up data were extracted from medical records. Physical status was assessed based on the following factors: age, gender, smoking history, American Society of Anesthesiologists (ASA) score, Charlson comorbidity index (CCI), presence or absence of chronic obstructive pulmonary disease (COPD), body mass index (BMI), and prognostic nutritional index (PNI). PNI was calculated as 10× serum albumin value +0.005× total lymphocyte count in peripheral blood just before resection.^15^ Tumor status was diagnosed according to the Japanese Gastric Cancer Association classification system.^16^

### 2.2 Surgical procedure

Extents of gastrectomy and lymph node dissection were defined based on tumor location and depth of invasion according to the Japanese Gastric Cancer Treatment Guidelines.^17^ D2 lymphadenectomy is recommended for tumors with more than clinical T2 or N1 disease, whereas D1+lymphadenectomy is allowable for clinical T1N0 tumors. D1 lymphadenectomy removed perigastric lymph nodes plus the lymph node at the base of the left gastric artery. D1+lymphadenectomy was defined as D1 lymphadenectomy plus nodal dissection around the celiac artery, along the common hepatic artery, and at the base of the splenic artery particularly for total and proximal gastrectomies. D2 lymphadenectomy was defined as D1+lymphadenectopmy plus nodal dissection along the proper hepatic artery, and around the distal splenic artery and splenic hilar with a splenectomy (particularly for total gastrectomy). For clinical stage I disease, laparoscopic gastrectomy was carried out.

### 2.3 Definition of pneumonia

Postoperative pneumonia was defined as higher than grade II on the Clavien‐Dindo classification.^18^ Pneumonia was diagnosed based on the findings of consolidation by chest X‐ray or computed tomography (CT), and on the clinical findings (ie presenting one or more of the following factors: temperature ≥38°C, cough productive of purulent sputum, and raised white cell count [≥10 000/mm^3^]).

### 2.4 Statistical analyses

We assessed the results of a univariate analysis of risk factors for postoperative pneumonia by using the Mann‐Whitney *U*‐test and Student's *t*‐test for continuous variables and with Pearson's chi‐squared test and Fisher's exact test for categorical variables as appropriate. Variables found to have a significant association with postoperative pneumonia in the univariate analysis were subjected to a multivariate logistic regression analysis. Cumulative survival rate was calculated by the Kaplan‐Meier method, and survival curves were compared using the log‐rank test. Cox proportional hazards model was constructed to identify independent prognostic factors. All statistical analyses were carried out using JMP statistical software (ver. 11: SAS, Cary, NC, USA). *P*‐values <.05 were considered significant.

## 3 RESULTS

### 3.1 Patient demographics and factors associated with pneumonia

Characteristics and surgical outcomes of the patients and a comparison by presence of postoperative pneumonia are shown in Tables 1 and 2. This study cohort contained 95 patients (38%) aged ≥80 years, with a mean age of 79.0±3.7 years. Postoperative pneumonia occurred in 32 patients (12.8%). Pneumonia was significantly associated with male gender (*P*=.027), presence of COPD (*P=*.016), and D2 lymphadenectomy (*P*<.001). Subsequent multivariate logistic regression analysis demonstrated that male gender (odds ratio [OR], 2.96; 95% confidence interval [CI], 1.13‐9.30; *P*=.026) and D2 lymphadenectomy (OR, 3.76; 95%CI, 1.72‐8.74; *P*<.001) were significant risk factors for the development of postoperative pneumonia (Table 3). Age, smoking history, ASA score, CCI, BMI, PNI, tumor stage, extent of gastrectomy, and operation time were not significantly associated with postoperative pneumonia.

**Table 1:** Postoperative pneumonia and patient demographics

Variable	All patients (n=250)	Postoperative pneumonia		*P*‐value
		Yes (n=32)	No (n=218)	
Age (years)				
Mean±SD	79.0±3.7	79.6±4.4	78.9±3.6	.342
75‐79	155 (62)	18 (56)	137 (63)	.473
≥80	95 (38)	14 (44)	81 (37)	
Gender				.027
Male	169 (68)	27 (84)	142 (65)	
Female	81 (32)	5 (16)	76 (35)	
Smoking history				.443
Current	46 (18)	4 (12)	42 (19)	
Ex‐smoker	94 (38)	15 (47)	79 (36)	
Never	110 (44)	13 (41)	97 (44)	
COPD				.016
Present	72 (29)	15 (47)	57 (26)	
Absent	178 (71)	17 (53)	161 (74)	
ASA score				.675
1	24 (10)	1 (3)	23 (11)	
2	172 (69)	25 (78)	147 (67)	
3	54 (22)	6 (19)	48 (22)	
CCI				.211
≤2	207 (83)	24 (75)	183 (84)	
≥3	43 (17)	8 (25)	35 (16)	
BMI (kg/m^2^) (mean±SD)	22.0±3.0	21.5±2.9	22.1±3.0	.391
PNI (mean±SD)	43.8±6.2	42.1±7.2	44.1±6.0	.098
Histological type				.617
Differentiated	166 (66)	20 (63)	146 (67)	
Undifferentiated	84 (34)	12 (37)	72 (33)	
Tumor stage				.212
I	145 (58)	14 (44)	131 (60)	
II	74 (30)	13 (41)	61 (28)	
III	31 (12)	5 (16)	26 (12)	

ASA, American Society of Anesthesiologists; BMI, body mass index; CCI, Charlson comorbidity index; COPD, chronic obstructive pulmonary disease; PNI, prognostic nutritional index; SD, standard deviation.

**Table 2:** Postoperative pneumonia and surgical outcomes

Variable	All patients (n=250)	Postoperative pneumonia		*P*‐value
		Yes (n=32)	No (n=218)	
Approach				.180
Open	161 (64)	24 (75)	137 (63)	
Laparoscopy	89 (36)	8 (25)	81 (37)	
Gastrectomy				.605
Total	83 (33)	12 (38)	71 (33)	
Distal or proximal	167 (67)	20 (62)	147 (67)	
Splenectomy				.082
No	219 (88)	25 (78)	194 (89)	
Yes	31 (12)	7 (22)	24 (11)	
Lymph node dissection				<.001
D1 or D1+	146 (58)	10 (31)	136 (62)	
D2	104 (42)	22 (69)	82 (38)	
Operation time (min), median (range)	303 (123‐702)	319 (184‐653)	298 (123‐702)	.971
Blood loss (mL), median (range)	257 (10‐3281)	374 (10‐2228)	246 (10‐3281)	.086
Year of operation				.428
2000‐2007	102 (41)	11 (34)	91 (42)	
2008‐2014	148 (59)	21 (66)	127 (58)	

**Table 3:** Multivariate analysis of risk factors for postoperative pneumonia

Variable	Odds ratio	95% CI	*P*‐value
Male gender	2.96	1.13‐9.30	.026
COPD	1.15	0.48‐2.61	.745
D2 lymph node dissection	3.76	1.72‐8.74	<.001

COPD, chronic obstructive pulmonary disease.

### 3.2 Relationship between pneumonia and survival

Operative mortality rate was 0.4% (one patient). Among the 250 patients during a median follow up of 30.5 (range 0‐157) months, 42 patients (17%) suffered recurrences, and 35 patients (14%) died from gastric cancer. Fifty patients (20%) died of non‐cancer‐related causes, including 13 (5%) with pneumonia, 11 (4%) of natural causes, six (2%) with cancer of another organ, five (2%) of unknown cause, four (2%) with heart disease, three (1%) with liver disease, and two (1%) with cerebral stroke.

**Figure 1:**
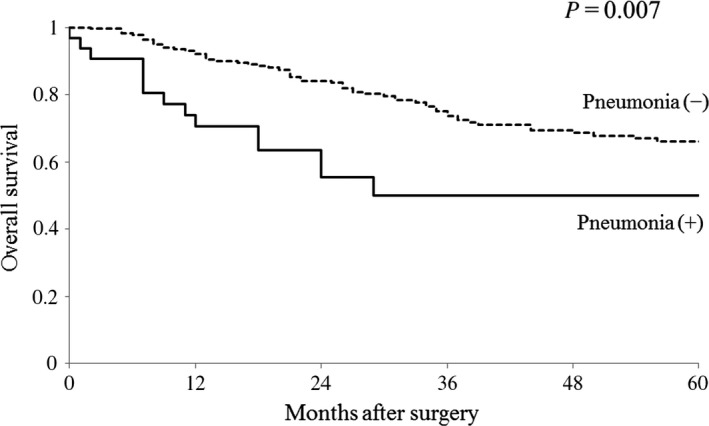
Kaplan‐Meier curves of overall survival for patients with or without pneumonia after curative gastrectomy

Patients with postoperative pneumonia had significantly shorter overall survival (OS) than those without pneumonia (log‐rank *P*=.007, Figure 1). Results of the Cox proportional hazards model of the factors that affected OS are shown in Table 4. Age (hazard ratio [HR] 1.90; 95% CI, 1.20‐2.99; *P*=.006), ASA score (HR 1.95; 95% CI, 1.17‐3.15; *P*=.011), type of gastrectomy (HR 2.41; 95% CI, 1.43‐4.01; *P*=.001), tumor stage (HR 1.70; 95% CI, 1.04‐2.78; *P*=.033) and postoperative pneumonia (HR 2.06; 95% CI, 1.05‐3.75; *P*=.036) were found to be independent prognostic factors.

**Table 4:** Cox proportional hazard model for overall survival after curative gastrectomy

Variable	Univariate analysis			Multivariate analysis		
	HR	95% CI	*P‐*value	HR	95% CI	*P‐*value
Age (≥80) years	2.02	0.32‐0.77	.002	1.90	1.20‐2.99	.006
Male gender	0.93	0.60‐1.49	.769			
ASA score (3)	1.67	1.02‐2.65	.042	1.95	1.17‐3.15	.011
CCI (≥3)	1.51	0.87‐2.50	.137			
BMI (<18.5)	0.94	0.46‐1.74	.861			
PNI (˂42)	1.63	1.06‐2.51	.027	1.33	0.83‐2.11	.235
pStage (II, III)	2.30	1.50‐3.57	<.001	1.70	1.04‐2.78	.033
Open approach	2.13	1.27‐3.83	.004	1.34	0.73‐2.57	.355
Total gastrectomy	2.73	1.78‐4.19	<.001	2.41	1.43‐4.01	.001
Splenectomy	2.05	1.18‐3.38	.012	1.27	0.66‐2.49	.477
Postoperative complication	1.40	0.90‐2.14	.133			
Severe complication (≥C‐D grade III)	1.72	0.57‐2.52	.761			
Infectious complication	1.60	1.00‐2.50	.052			
Intra‐abdominal infectious complication	1.48	0.76‐2.62	.235			
Pneumonia	2.19	1.18‐3.79	.015	2.06	1.05‐3.75	.036

ASA, American Society of Anesthesiologists; BMI, body mass index; CCI, Charlson comorbidity index; C‐D, Clavien‐Dindo; PNI, prognostic nutritional index.

### 3.3 Comparison of survival between the extents of lymph node dissection

**Figure 2:**
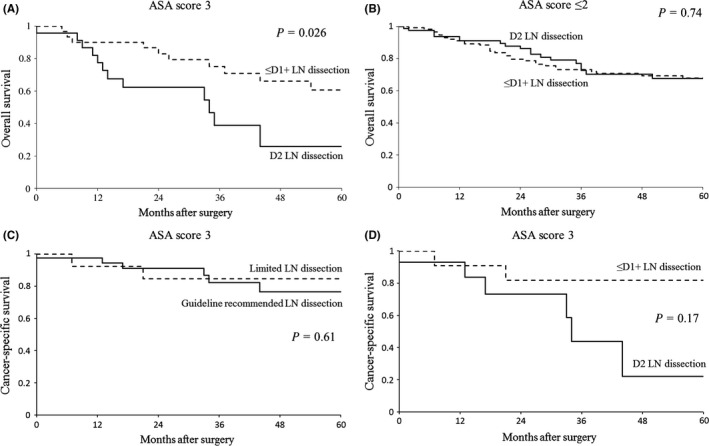
Kaplan‐Meier curves for patients stratified by American Society of Anesthesiologists (ASA) score according to extent of lymph node dissection. (A) Overall survival (OS) of patients with ASA score 3 (n=54). (B) OS of patients with ASA score ≤2 (n=196). (C) Cancer‐specific survival (CSS) of patients with ASA score 3 (n=54). (D) CSS of patients with ASA score 3 with node‐positive or T2‐T4 tumors (n=26)

To determine which patients were unfit for D2 lymphadenectomy, we compared the OS curve stratified by ASA score between D2 lymphadenectomy and D1 or D1+lymphadenectomy. D2 lymphadenectomy significantly decreased OS rate compared to D1 or D1+lymphadenectomy in the 54 elderly patients who had an ASA score of 3 (log rank *P*=.026, Figure 2A), whereas D2 lymphadenectomy did not influence OS negatively in 196 patients with an ASA score ≤2 (Figure 2B). Regarding patients with ASA score 3, significant differences between guideline recommended lymph node dissection and less than recommended lymph node dissection were not found in the cancer‐specific survival (CSS) rate of all 54 patients, and in the CSS of the 26 patients with node‐positive or T2‐T4 tumors during R0 resection (Figure 2C,D).

## 4 DISCUSSION

Several studies have shown a relationship between postoperative complications and long‐term survival in various malignancies.^10^, ^12^, ^19^, ^20^ Regarding gastric cancer, infectious complications after gastrectomy caused by anastomotic leakage, procedure‐related infection or any type of infection were reported to have a negative effect on survival.^21^, ^22^, ^23^ For elderly patients, severe postoperative complications were reported to be associated with worse clinical outcomes.^14^ However, these investigations did not focus solely on the impact of postoperative pneumonia on long‐term survival. In the present study, we found that postoperative pneumonia had an adverse impact on overall survival in elderly patients undergoing curative‐intent gastrectomy, and our analysis showed that postoperative pneumonia was also an independent predictor for worse survival. These findings mean that postoperative pneumonia affects prognosis separately from preoperative health status, operative procedure, and disease stage in elderly patients.

A potential explanation for these findings is that postoperative pneumonia prolongs patient hospital stay, worsens postoperative disability, and reduces activities of daily living. Impaired physical status may make elderly patients more susceptible to cancer‐related death and cancer‐unrelated death. An additional possible explanation is host immunosuppression induced by inflammatory responses to complications. As an inflammatory disease, pneumonia leads to immunomodulatory impairment. These circumstances may facilitate the progression of residual microscopic disease that manifest as a recurrence or death. Further investigations such as nationwide studies are necessary to elucidate the mechanisms by which postoperative complications worsen prognoses.

Frequency of pneumonia after gastrectomy in elderly patients aged ≥75 years has been reported as 5.1‐13.3%, which is significantly higher than that of younger patients (ie 45‐65 years old).^14^, ^24^, ^25^ Pneumonia developed in 12.8% of the present patients, which is similar to findings of previous studies, but advanced age (≥80 years) did not increase the incidence of pneumonia in our study cohort. One possible reason for this result is that we chose patients ≥75 years age, which is a heterogeneous cohort with substantial differences in health status, and thus the age‐related changes do not necessarily correspond to chronological age. Reported risk factors for the development of postoperative pneumonia in patients aged ≥75 years are gender, ASA‐PS, PS (performance status), impaired respiratory function, diabetes mellitus and blood transfusion, but these factors varied among all three studies.^14^, ^24^, ^25^ This is likely to be a reflection of the small sample size in each study.

The present study's multivariate analysis identified radical lymphadenectomy as an independent risk factor for the development of postoperative pneumonia. Gastrectomy with D2 lymphadenectomy has been a standard procedure for gastric cancer, but there is no available prospective study which confirms the feasibility of D2 lymphadenectomy for patients >75 years old. Karasaki et al.^26^ demonstrated that the extent of lymph node dissection had no influence on cancer death in patients aged ≥80 years. Takeshita et al.^27^ reported that radical lymphadenectomy in patients aged ≥80 years may reduce life expectancy, although modified lymphadenectomy had little effect on disease‐specific survival. With respect to the effect of radical lymphadenectomy on postoperative physical status, an exhaustive disruption of the plexus of sympathetic nerves surrounding the celiac, common hepatic and splenic arteries by radical lymphadenectomy is considered a cause of diarrhea, diminished eating habits and esophageal reflex, which have the potential to cause aspiration pneumonia as a lasting postoperative sequela. These postoperative disabilities impair patients’ physical status. Ichikura et al.^28^ reported that limited celiac dissection could improve patients’ physical condition postoperatively.

To modulate the extent of lymph node dissection on the basis of curative intent and physical fitness, we stratified the elderly patients according to ASA grade to identify the patients who were not fit for radical lymphadenectomy. We found that radical lymphadenectomy was associated with a poorer overall survival of the patients who had higher ASA scores. Limited lymph node dissection seems not to influence the CSS in ASA score 3 elderly patients. Inferiority of less than D2 lymphadenectomy for frail patients with advanced stage tumor was not found in comparison with D2 lymphadenectomy in CSS. Considering postoperative complications and impairment, the survival benefit of radical lymphadenectomy for frail elderly patients remains to be defined. Given the long‐term adverse effects of postoperative pneumonia, surgeons need to plan the extent of lymph node dissection cautiously to prevent pneumonia and to improve long‐term outcomes according to patients’ frailty.

This retrospective study at a single institution had some limitations. Our study included diverse patients with various physical conditions and disease status. The frailer patients tended to undergo less‐invasive procedures in clinical practice and, consequently, their physical status may not reflect the development of postoperative pneumonia. Confirmation of these findings requires a larger study, but our results provide a strategic concept for gastric cancer surgery in elderly patients.

In conclusion, postoperative pneumonia is responsible for worsening long‐term survival of elderly patients undergoing curative‐intent gastrectomy. Curability should be maintained. Extent of lymph node dissection during curative resection might be limited to prevent postoperative pneumonia, based on the patient's frailty. A limited lymphadenectomy during curative resection should be considered for the goal of preventing postoperative pneumonia in frail elderly gastric cancer patients with ASA score 3.

## DISCLOSURE

Conflict of Interest: Authors declare no conflicts of interest for this article.

Approval of the Research Protocol: This retrospective research project was approved by Institutional Review Board of Kobe University, and it conforms to the provisions of the Declaration of Helsinki.

Author Contribution: Substantial contribution to the conception or design of the work, or acquisition, analysis or interpretation of data for the work: Y. Matsuda, M. Yamamoto, H. Hasegawa, K. Yamashita, T. Matsuda and Y. Sumi. Drafting the work or revisiting it critically for important intellectual content: S. Suzuki and S. Kanaji. Final approval of the version to be published: Y. Kakeji. Agreement to be accountable for all aspects of the work in ensuring that questions related to the accuracy or integrity of any part of the work are appropriately investigated and resolved: T. Oshikiri and T. Nakamura.
